# The risk factors of progestational anxiety, depression, and sleep disturbance in women with recurrent pregnancy loss: A cross-sectional study in China

**DOI:** 10.3389/fpsyg.2023.1116331

**Published:** 2023-03-31

**Authors:** Ting-ting Wang, Yi-lin Liu, Yue Hou, Jia-po Li, Chong Qiao

**Affiliations:** ^1^Department of Obstetrics and Gynecology, Shengjing Hospital of China Medical University, Shenyang, China; ^2^Key Laboratory of Obstetrics and Gynecology of Higher Education of Liaoning Province, Shenyang, China

**Keywords:** recurrent pregnancy loss, anxiety, depression, sleep quality, sleep disturbance

## Abstract

**Background:**

The risk factors of progestational anxiety, depression, and sleep disturbance in women with a history of recurrent pregnancy loss (RPL) remain controversial, additional study is needed to investigate the incidence and risk factors of progestational anxiety, depression, and sleep quality in RPL women.

**Methods:**

A cross-sectional study was conducted among 663 non-pregnant RPL women in Northeast China from October 2019 to July 2022. We assessed the state of anxiety, depression, and sleep quality before pregnancy using structured questionnaires, including sociodemographic characteristics, state-trait anxiety scale (STAI), center for epidemiological survey, depression scale (CES-D), Pittsburgh sleep quality index (PSQI), and symptom self-rating scale (SCL-90). Logistic regression was used to evaluate the association between sleep quality and anxiety, depression. Pearson’s correlation was used to evaluate the correlation between anxiety and depression. Multivariate logistic regression analysis was used to find the risk factors of depression symptoms. The receiver operating characteristic curve (ROC) was used to evaluate the predictive value of the model.

**Results:**

The incidence of state anxiety, trait anxiety, depression, and sleep disturbance in RPL women were 60.3, 51.7, 33.9, and 31.2%, respectively. The level of anxiety and depression in RPL women varied at different stages of treatment. In a longitudinal study (25 pairs), we found the level of state anxiety and trait anxiety were significantly lower after the cause was identified. Sleep disturbance is positively correlated with anxiety and depression. Logistic regression showed that the number of miscarriages ≥4 (Odds ratio (OR) = 2.268, 95%CI 1.300–3.956), Low household family income (OR = 1.613, 95%CI 1.036–2.513/OR = 2.361, 95%CI 1.095–5.092), interval since last miscarriage <6 months (OR = 2.154, 95%CI 1.246–3.726) and sleep disturbance (OR = 5.523, 95%CI 3.542–8.614) were associated with the occurrence of depressive symptoms. At the same time, anxiety can be used as a predictor of depression.

**Conclusion:**

Recurrent pregnancy loss women have a certain degree of anxiety, depression, and sleep disturbance. Education level, interval since the last miscarriage <6 months, and sleep disturbance are risk factors for anxiety and depression. A history of pregnancy loss after 14 weeks and no living birth are also closely related to anxiety. Therefore, it is necessary to pay close attention to the psychological state of RPL women and provide appropriate psychosocial support to reduce the occurrence of negative emotions.

## Introduction

Recurrent pregnancy loss (RPL) was defined as 2 or more consecutive pregnancy losses (Medicine, 2020). It affects 2–5% of women worldwide and is an important reproductive health issue (El Hachem et al., 2017). A pregnancy loss can be both physically and psychologically a traumatic experience for women and their families.

Chinese people traditionally consider fertility and childbearing ability of great importance to women. At the same time, there is a tendency in society to think that not having children is always the woman’s fault (Fu et al., 2015). The etiology of RPL is complex and the screening process is lengthy, so it takes a long time to start treatment before the etiology is identified. Therefore, RPL women are prone to anxiety and depression under the dual effect of physical and psychological damage and pressure from family and the outside world. In recent years, with the transformation from the traditional medical model to a bio-psycho-social medical model, more and more attention has been paid to the psychological factors of RPL women.

An article of previous miscarriage-related literature shows that almost half the women had features of depression disorder after pregnancy loss (Kulathilaka et al., 2016). Repetitious miscarriages cause women to suffer from both psychological and physical stress, which in turn increases the risk of subsequent miscarriage (Wang et al., 2021). Anxiety and depression were highly prevalent in pregnant women with a history of recurrent miscarriage, especially in early pregnancy, and then decreased (Qu et al., 2021). Anxiety and depression act on the hypothalamic–pituitary–adrenal axis to release endocrine hormones. Abnormally activated HPA axis and high CRH level will not only lead to increased risk of obstetric complications such as eclampsia (Kurki et al., 2000) and preterm birth (Littleton et al., 2007), but also be associated with cognitive dysfunction in infants (Adamson et al., 2018).

Half of the etiology of RPL is still unknown, which undoubtedly increases the psychological burden of women with RPL and leads to the emergence of adverse psychological problems. Most researches on psychological problems in RPL were small in sample sizes and varied in psychological assessment scale, thereby making the results rather mixed. The prevalence of depression in reported studies ranged from 8.6–37%, while anxiety 7–45% (Craig et al., 2002; Kolte et al., 2015; He et al., 2019; Gao et al., 2020). And few studies have explored the presence of sleep disturbance in RPL women. Also, no studies have explored RPL women’s psychological change during the etiological screening phase and the initiation of treatment. Therefore, our research group conducted a cross-sectional study of RPL women in China. In this paper, multiple scales combined with sociodemographic and obstetric factors were used for psychological assessment of RPL women. First of all, we investigated the incidence of anxiety and depression in RPL women, then compared the change trend during the etiological screening phase and the initiation of treatment. We explored the association between sleep quality and anxiety, depression, the correlation between anxiety and depression, and finally explored the related factors of anxiety and depression. This helps to find out the possible psychological problems of RPL women as early as possible and provide scientific guidance.

## Materials and methods

### Study design and subjects

A cohort basing on recurrent pregnancy loss population in Shengjing Hospital Affiliated to China Medical University located in northeast China was established to study the effects of pre-pregnancy and gestational exposure on pregnancy outcomes from October 2019 to July 2022. Subjects with a history of depression, anxiety, and other psychological problems or currently using psychotropic drugs or refusing to participate were excluded. A total of 1,261 questionnaires were collected, 133 were excluded due to pregnancy, 267 patients were excluded due to incomplete sociodemographic information, and a total of 861 subjects were finally included, with an effective response rate of 74.1%. According to the number of pregnancy losses, they were divided into three groups: Group 1 represents subjects with RPL (663 cases): ①spontaneous miscarriage for two or more consecutive times. ②20–45 years old. ③No history of cognitive impairment or mental illness. ④Did not take any psychotropic drugs. ⑤Willing to accept investigators. Exclusion criteria: ①illiteracy. ②Known mental illness and major medical and surgical complications. ③Long-term use of psychotropic drugs. ④ Refuse to accept the investigators. Group 2 represents subjects with a history of one pregnancy loss (124 cases): ①a history of one spontaneous miscarriage. ②20–45 years old. ③No history of cognitive impairment or mental illness. ④Did not take any psychotropic drugs. ⑤Willing to accept investigators. Exclusion criteria are the same as the RPL group. And group 3 represents subjects with no previous miscarriage (74 cases): ①no history of previous pregnancy loss. ②20–45 years old. ③No history of cognitive impairment or mental illness. ④Did not take any psychotropic drugs. ⑤Willing to accept investigators. Exclusion criteria are the same as the RPL group.

This study included a small longitudinal study in which 25 of the subjects completed two questionnaires before and after identifying the cause.

The whole pregnancy is clinically divided into three periods, the first trimester occurs less than 14 weeks of gestation, the second trimester occurs between 14 and 28 weeks of gestation and the third trimester of pregnancy occurs after 28 weeks. Most miscarriages occur in the first trimester, and the psychological state of early and late miscarriage may be different. Therefore, 14 weeks was taken as the critical point in this study to investigate the influence of a history of pregnancy loss after 14 weeks on the level of anxiety and depression.

The study was approved by the Ethics Review Board of Shengjing Hospital Affiliated to China Medical University (approval number 2018PS381K). All subjects were guaranteed anonymity during data processing and informed consent was obtained.

### Measures

After obtaining informed consent from the subjects, a questionnaire was used to collect information from all included subjects. All included subjects completed the questionnaire at the first pre-pregnancy visit, and some of them completed the same questionnaire again at the initial stage of treatment after the etiology was identified.

The questionnaire mainly includes:

#### State-trait anxiety inventory

It consists of two subscales assessing two different types of anxiety, with a total of 40 questions to assess the current anxiety mode and personality anxiety tendencies. The State Anxiety Scale, S-AI, reflects the severity of the subject’s current anxiety symptoms, and the Trait Anxiety Scale, T-AI, reflects the subject’s consistent or usual anxiety. All scores ranged from 20 to 80. Higher scores indicate higher levels of anxiety. A threshold score of 40 is considered a clinically meaningful threshold value for identifying state anxiety, trait anxiety (Julian, 2011). The Chinese version of the STAI has demonstrated good reliability and validity (Du et al., 2022).

#### Center for epidemiological survey, depression scale

The CESD consists of 40 questions that respond to feelings or behaviors that may have occurred in the past week. A CESD score of ≥16 is often used as a threshold for clinical depression (Smarr and Keefer, 2011). The Chinese version of the CES-D is widely used, with the internal consistency of 0.855 and test–retest reliability of 0.91 in primary care patients, and the Cronbach’s alpha of 0.91 in Chinese adolescents (Jiang et al., 2019; Ling et al., 2021).

#### Pittsburgh sleep quality index

It consists of 7 subscales assessing sleep quality, time to fall asleep, sleep duration, sleep efficiency, sleep disorders, hypnotic drug use, and daytime dysfunction. Each subscale has a score between 0 and 3, with an overall score of 0–21. The higher the score, the worse the sleep quality. The current study uses an established threshold of scores above 5 to identify poor sleep quality (Buysse Dj et al., 1989). The Chinese version of the PSQI has good psychometric properties (Yan et al., 2021).

#### Symptom checklist 90-R

It consists of nine subscales (90 items): somatization, obsessive–compulsive symptoms, interpersonal sensitivity, depression, anxiety, hostility, phobia, paranoia, and psychosis. The SCL-90 scale has good discrimination between individuals with psychological symptoms (i.e., at risk for psychological disorder or borderline psychological disorder; Zanarini et al., 2003; Nickel et al., 2006). A total score of more than 160, or more than 43 items scored ≥2, or the average score of any subscale >2, needs to be considered a screening positive and requires further examination. The Chinese version of the SCL-90-R was validated and displayed high internal consistency (Yu et al., 2020).

#### Socio-demographic characteristics and obstetric questionnaire

Age and education levels of the couple, height and weight (to calculate the body mass index, BMI), duration of marriage, monthly household income, interval since the last miscarriage, number of previous miscarriage, pregnancy loss after 14 weeks, and living birth.

### Statistical analysis

Data were statistically analyzed using the statistical software SPSS 26.0. Continuous variables were expressed as mean ± standard deviation, while categorical variables as the number of cases (percentage). *T*-test, one-way analysis of variance (ANOVA) in combination with LSD posthoc tests were used to compare with continuous variables, Chi-square (*χ*^2^) tests were used for the comparison of categorical variables, and unpaired t-tests for continuous variables. The longitudinal study used repeated measure design *T*-test. We analyze the correlation between anxiety and depression in Pearson. Logistic regression was used to adjust for possible confounding factors by including variables to be analyzed and corrected. Binary logistic regression analysis was performed to explore possible predictors for anxiety and depression in RPL women, and the resulting predicted probabilities were analyzed by ROC curves. A *p*-value of <0.05 was considered statistically significant.

## Results

### Comparison of social characteristics and obstetric characteristics of subjects

The basic characteristics of the study subjects for each group were summarized in Table 1. No significant difference was observed between the two groups in age, BMI, monthly household income, education attainment of females and males and duration of marriage (*p* > 0.05).

**Table 1:** Comparison of social characteristics and obstetric characteristics of subjects.

Variables	Group 1 (*n* = 663)	Group 2 (*n* = 124)	Group 3 (*n* = 74)	*P*-value
Age (mean ± SD)	32.83 ± 4.053	32.97 ± 4.088	33.07 ± 4.514	ns
BMI (mean ± SD)	23.13 ± 3.521	23.14 ± 3.647	22.63 ± 4.058	ns
Education of women (*n*, %)				ns
Primary and junior secondary	81(12.2)	2(1.6)	4(5.4)	
Senior secondary and junior college	174(26.2)	10(8.1)	44(59.5)	
Bachelor’s degree or above	408(61.5)	112(90.3)	26(35.1)	
Education of men (*n*, %)				ns
Primary and Junior Secondary	95(14.3)	4(3.2)	3(4.1)	
Senior Secondary and Junior College	214(32.3)	42(33.9)	29(39.2)	
Bachelor’s degree and above	354(53.4)	78(62.9)	42(56.8)	
Monthly household income (*n*, %)				ns
<5,000	55(8.3)	10(8.1)	1(1.4)	
5,000–9,999	281(42.4)	47(37.9)	35(47.3)	
≥10,000	327(49.3)	67(54.0)	38(51.4)	
Duration of marriage (year, mean ± SD)	5.006 ± 3.613	4.854 ± 3.714	5.528 ± 4.560	ns

Group 1: RPL women; Group 2: women with one history of pregnancy loss; Group 3: women without history of previous pregnancy loss.

BMI, body mass index.

^*^P < 0.05; ^**^P < 0.01; ^***^P < 0.001.

### Comparison of levels of anxiety, depression, and psychiatric symptoms between RPL women and non-RPL women

As shown in Table 2, except for sleep quality (PSQI), the anxiety (S-AI, T-AI), depression (CES-D), and psychiatric symptoms (SCL-90) scores of RPL women were higher than those two groups (*p* < 0.05). The SCL-90 subscales were further compared, including somatization, obsessive–compulsive disorder, interpersonal sensitivity, depression, anxiety, hostility, phobia, paranoia, and psychosis. Eight of these psychiatric symptoms scores were significantly higher in the RPL women than in the comparison group (e.g., obsessive–compulsive disease, interpersonal sensitivity, depression, anxiety, hostility, phobia, paranoia, and psychosis). And the incidence of state anxiety, trait anxiety, and depression in RPL women was 60.3, 51.7, and 33.9%, respectively.

**Table 2:** Comparison of levels of anxiety, depression and psychiatric symptoms between recurrent pregnancy loss (RPL) women and non-RPL women.

Scales	Group 1 (*n* = 663)	Group 2 (*n* = 124)	Group 3 (*n* = 74)	*F*	*P*-value
S-AI	42.22 ± 11.060	40.74 ± 11.623	37.59 ± 9.677	6.294	0.002^**^
T-AI	40.32 ± 10.421	38.94 ± 11.015	36.18 ± 9.755	5.684	0.004^**^
CES-D	12.76 ± 8.631	11.69 ± 8.921	10.22 ± 6.799	3.432	0.033^*^
PSQI	4.63 ± 2.771	4.60 ± 2.653	4.47 ± 2.834	0.114	0.893
SCL-90	126.81 ± 33.081	121.19 ± 26.337	113.64 ± 22.665	6.843	0.001^**^
Somatization	16.43 ± 4.128	16.30 ± 4.106	15.54 ± 4.031	1.542	0.214
Obsessive–compulsive disease	16.21 ± 4.996	15.76 ± 4.471	14.54 ± 4.208	4.097	0.017^*^
Interpersonal sensitivity	12.83 ± 4.182	11.85 ± 3.185	11.15 ± 2.683	8.313	<0.001^***^
Depression	19.01 ± 6.373	17.82 ± 5.030	16.64 ± 4.183	6.455	0.002^**^
Anxiety	14.11 ± 4.402	13.75 ± 3.756	12.72 ± 3.009	3.799	0.023^*^
Hostility	8.76 ± 2.978	8.10 ± 2.373	7.58 ± 1.672	7.891	<0.001^***^
Phobia	8.67 ± 2.610	8.19 ± 1.727	7.93 ± 1.465	4.538	0.011^*^
Paranoia	7.84 ± 2.294	7.43 ± 1.947	6.82 ± 1.328	8.239	<0.001^***^
Psychosis	13.22 ± 3.661	12.35 ± 2.711	11.74 ± 2.341	8.411	<0.001^***^
Additional symptoms	9.74 ± 2.715	9.65 ± 2.430	8.97 ± 2.100	2.840	0.059

Group 1: RPL women; Group 2: women with one history of pregnancy loss; Group 3: women without history of previous pregnancy loss.

S-AI, state anxiety; T-AI, trait anxiety; CES-D, depression; PSQI, sleep quality; SCL-90, psychiatric symptoms.

^*^P < 0.05; ^**^P < 0.01; ^***^P < 0.001.

### Comparison of levels of anxiety and depression at different diagnosis and treatment stages in RPL women

Among the 663 RPL women who participated in the study, 663 completed the questionnaire for the first visit before pregnancy, and only 25 completed the questionnaire for treatment after finding out the cause. The scale scores of the patients were compared between the first visit before pregnancy and the treatment after finding out the cause, as shown in Table 3. Both state anxiety and trait anxiety levels were significantly lower after the etiology was identified than at the first visit (*p* = 0.003, *p* = 0.009).

**Table 3:** Comparison of levels of anxiety and depression at different diagnosis and treatment stages in recurrent pregnancy loss (RPL) women (*n* = 25).

Scales	Screening for etiology	Under treatment	*t*	*P*-value
S-AI	42.65 ± 10.976	35.08 ± 10.677	3.280	0.003^**^
T-AI	42.19 ± 10.365	35.77 ± 11.864	2.842	0.009^**^
CES-D	12.08 ± 8.400	12.04 ± 9.962	0.029	0.977
PSQI	6.12 ± 2.732	5.50 ± 2.159	1.150	0.261
SCL-90	127.65 ± 34.285	123.62 ± 43.521	0.862	0.397

S-AI, state anxiety; T-AI, trait anxiety; CES-D, depression; PSQI, sleep quality; SCL-90, psychiatric symptoms.

^*^P < 0.05; ^**^P < 0.01; ^***^P < 0.001.

### Association between sleep quality and anxiety, depression symptoms

The mean score of PSQI was 4.63 ± 2.771, indicating that RPL women had a good overall sleep quality. 31.2% had sleep quality disturbance (27.5% mild, 3.6% moderate, and 0.1% severe). Because only one patient had poor sleep quality, thus we combined the women with PSQI scores of 6–10, and 11–21 into one group. Sleep quality is negatively correlated with anxiety and depression. As shown in Table 4, women with mild sleep disturbance had a higher odds for state anxiety (OR = 2.884, 95%CI 1.956–4.252), trait anxiety (OR = 3.258, 95%CI 2.251–4.715) and depressive symptoms (OR = 5.729, 95%CI 3.946–8.317). While women with moderate to severe sleep disturbance whose state anxiety (OR = 3.568, 95%CI 1.317–9.682) and trait anxiety (OR = 4.238, 95%CI 1.662–10.811) and depression symptoms (OR = 11.875, 95%CI 4.615–30.554) were much higher.

**Table 4:** Association between sleep quality and anxiety, depression symptoms (*n* = 663).

PSQI^#^	0–5 No. of cases (%)	6–10 No. of cases (%)	11–21 No. of cases (%)
**Having state-anxiety symptoms**
No (*n* = 263)	215(47.1)	28(15.4)	20(80.0)
Yes (*n* = 400)	241(52.9)	154(84.6)	5(20.0)
OR (95%CI)	Ref	2.884 (1.956–4.252)^**^	3.568 (1.317–9.682)^*^
**Having trait-anxiety symptoms**
No (*n* = 320)	261(57.2)	40(22.0)	19(76.0)
Yes (*n* = 343)	195(42.8)	142(78.0)	6(24.0)
OR (95%CI)	Ref	3.258 (2.251–4.715)^**^	4.238 (1.662–10.811)^**^
**Having depression symptoms**
No (*n* = 438)	360(78.9)	59(32.4)	19(76.0)
Yes (*n* = 225)	96(21.1)	123(67.6)	6(24.0)
OR (95%CI)	Ref	5.729 (3.946–8.317)^**^	11.875 (4.615–30.554)^**^

PSQI represents sleep quality ^*^P < 0.05; ^**^P < 0.01; ^***^P < 0.001.

# Level of sleep quality: 0–5 good sleep quality; 6–10 mild sleep disturbance; 11–21 moderate to severe sleep disturbance.

### Risk factors for state anxiety, trait anxiety and depression in RPL women

In this study, multiple scales were used to evaluate anxiety and depression. S-AI, T-AI, and SCL-90 anxiety subscales were used to evaluate anxiety, and CES-D and SCL-90 depression subscales were used to evaluate depression. Correlation analysis revealed that there was a positive correlation between each dimension of anxiety scale and each scale of depression, as illustrated in Table 5.

**Table 5:** Pearson’s correlation between anxiety and depression.

Scales	S-AI	T-AI	SCL90-A	CES-D	SCL90-D
S-AI	1	0.876^**^	0.519^**^	0.640^**^	0.522^**^
T-AI	0.876^**^	1	0.554^**^	0.671^**^	0.586^**^
SCL90-A	0.519^**^	0.554^**^	1	0.639^**^	0.846^**^
CES-D	0.640^**^	0.671^**^	0.639^**^	1	0.733^**^
SCL90-D	0.522^**^	0.586^**^	0.846^**^	0.733^**^	1

S-AI, state anxiety; T-AI, trait anxiety; CES-D, depression; PSQI, sleep quality; SCL90-A, anxiety subscale in SCL-90; SCL90-D, depression subscale in SCL-90.

^*^P < 0.05; ^**^P < 0.01; ^***^P < 0.001.

Multiple regression analysis using the step-wise method was conducted. As noted in Tables 6, 7, in the logistic regression model for predicting state anxiety symptoms, education level (OR = 1.855, 95%CI 1.096–3.138/OR = 1.527, 95%CI 1.041–2.239), interval since last miscarriage <6 months (OR = 2.149, 95%CI 1.342–3.443), pregnancy loss >14 weeks (OR = 1.599, 95%CI 1.020–2.506), sleep disturbance (OR = 2.951, 95%CI 2.023–4.305) were closely related to state anxiety. While education level (OR = 2.937, 95%CI 1.723–5.005/OR = 1.487, 95%CI 1.024–2.162), interval since last miscarriage <6 months (OR = 1.598, 95%CI 1.028–2.484), sleep disturbance (OR = 3.540, 95%CI 2.465–5.084) and live birth (OR = 0.419, 95%CI 0.205–0.847) were closely related to trait anxiety. Similarly, after adjusting the related confounders, six variables were retained in the logistical regression model for predictors of depression symptoms.

**Table 6:** Risk factors for state anxiety (S-AI scores) identified by multivariable logistic regression analysis (*n* = 663).

S-AI	No. of cases/no. Total cases (%)	Univariate OR (95%CI)	*P*-value	Adjusted OR (95%CI)^#^	*P*-value
**Age**
≤29	81/128	Reference			
30–35	218/374	0.811(0.536–1.227)	0.321		
≥36	101/161	0.977(0.604–1.580)	0.924		
**BMI**
<18.5	23/32	Reference			
18.5–24	244/416	0.555(0.251–1.229)	0.147		
≥24	133/215	0.635(0.280–1.439)	0.276		
**Education**
Primary and Junior Secondary	55/81	1.621(0.977–2.688)	0.061	1.855(1.096–3.138)	0.030^*^
Senior Secondary and Junior College	114/174	1.456(1.007–2.105)	0.046^*^	1.527(1.041-2.239)	0.001^**^
Bachelor’s degree or above	231/408	Reference		Reference	
**Length of marriage**
<3	105/163	Reference			
3–5	167/278	0.831(0.557–1.240)	0.365		
>5	128/222	0.752(0.496–1.141)	0.180		
**Monthly household income**
<5,000	32/55	1.042(0.584–1.858)	0.890		
5,000–9,999	181/281	1.355(0.976–1.881)	0.069		
≥10,000	187/327	Reference			
**Pregnancy loss**
2–3	326/554	Reference			
≥4	74/109	1.479(0.956–2.287)	0.079		
**Interval since last miscarriage**
<6	79/109	1.911(1.215–3.006)	0.005^**^	2.149(1.342–3.443)	0.001^**^
≥6	321/554	Reference		Reference	
**Pregnancy loss after 14 weeks**
Yes	80/117	1.680(1.092–2.584)	0.018^*^	1.599(1.020-2.506)	0.041^*^
No	322/546	Reference		Reference	
**Live birth**
Yes	22/41	0.747(0.396–1.410)	0.369		
No	378/622	Reference			
**PSQI**
<6	241/456	2.955(2.038–4.285)	<0.001^***^	2.951(2.023–4.305)	<0.001^***^
≥6	159/207	Reference		Reference	
**Active smoking**
Yes	37/59	1.117(0.643–1.940)	0.696		
No	363/604	Reference			
**Passive smoking**
Yes	161/273	0.908(0.662–1.245)	0.550		
No	239/390	Reference			
**Alcohol drinking**
Yes	63/101	1.107(0.715–1.713)	0.648		
No	337/562	Reference			

# Adjusting age, BMI, duration of marriage, smoking status, and drinking status.

^*^P < 0.05; ^**^P < 0.01; ^***^P < 0.001.

**Table 7:** Risk factors for trait anxiety (T-AI scores) identified by multivariable logistic regression analysis (*n* = 663).

T-AI	No. of cases/no. Total cases (%)	Univariate OR (95%CI)	*P*-value	Adjusted OR (95%CI)^#^	*P*-value
**Age**
≤29	65/128	Reference			
30–35	193/374	1.033(0.692–1.544)	0.872		
≥36	85/161	1.084(0.681–1.725)	0.734		
**BMI**
<18.5	23/32	Reference			
18.5–24	210/416	0.399(0.180–0.883)	0.023^*^		
≥24	110/215	0.410(0.181–0.927)	0.032^*^		
**Education**
Primary and Junior Secondary	54/81	2.250(1.363–3.714)	0.002^**^	2.937(1.723–5.005)	<0.001^***^
Senior Secondary and Junior College	97/174	1.417(0.992–2.025)	0.055	1.487(1.024–2.162)	0.037^*^
Bachelor’s degree or above	192/408	Reference		Reference	
**Length of marriage**
<3	89/163	Reference			
3–5	139/278	0.831(0.564–1.225)	0.351		
>5	115/222	0.894(0.596–1.340)	0.587		
**Monthly household income**
<5,000	33/55	1.624(0.908–2.905)	0.102		
5,000–9,999	153/281	1.294(0.940–1.782)	0.114		
≥10,000	157/327	Reference			
**Pregnancy loss**					
2–3	276/554	Reference			
≥4	67/109	1.607(1.056–2.446)	0.027^*^		
**Interval since last miscarriage**
<6	278/554	1.467(0.966–2.226)	0.072	1.598(1.028–2.484)	0.037^*^
≥6	65/109	Reference		Reference	
**Pregnancy loss after 14 weeks**
Yes	71/117	1.555(1.035–2.336)	0.034^*^		
No	272/546	Reference			
**Live birth**
Yes	15/41	0.517(0.269–0.995)	0.048^*^	0.419(0.205-0.857)	0.017^*^
No	328/622	Reference		Reference	
**PSQI**
<6	195/456	3.357(2.356–4.785)	<0.001^***^	3.540(2.465–5.084)	<0.001^***^
≥6	148/207	Reference		Reference	
**Active smoking**
Yes	33/59	1.204(0.703–2.062)	0.499		
No	310/604	Reference			
**Passive smoking**
Yes	138/273	0.922(0.677–1.257)	0.609		
No	205/390	Reference			
**Alcohol drinking**
Yes	53/101	1.036(0.678–1.583)	0.871		
No	290/562	Reference			

# Adjusting age, BMI, duration of marriage, smoking status, and drinking status.

^*^P < 0.05; ^**^P < 0.01; ^***^P < 0.001.

**Figure 1:**
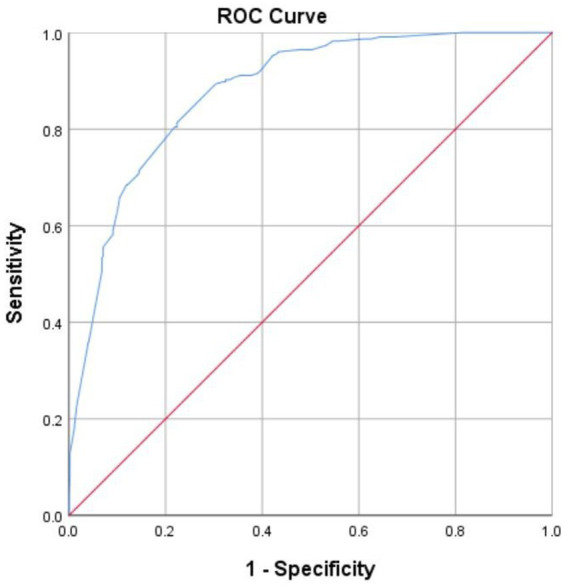
Receiver operating characteristic curve (ROC) of a predictive model of depression in recurrent pregnancy loss (RPL) women.

The predictors of depressive symptoms were listed in Table 8. The number of miscarriages ≥4 (OR = 2.268, 95% CI 1.300–3.956), Low monthly household income (OR = 1.613, 95%CI 1.036–2.513/OR = 2.361, 95%CI 1.095–5.092), interval since the last miscarriage <6 months (OR = 2.154, 95%CI 1.246–3.726) and sleep disturbance (OR = 5.523, 95%CI 3.542–8.614) were associated with the occurrence of depressive symptoms. At the same time, state anxiety (OR = 2.466, 95%CI 1.282–4.744) and trait anxiety (OR = 8.925, 95%CI 4.911–16.220) could predict depression symptoms. According to the Logistic regression model, the prediction probability was obtained, and whether CES-D score was ≥16 was used as the state variable to draw the ROC curve of the model. The area under the ROC curve (AUC) predicted by the model for depression in RPL women was 0.878 (95%CI: 0.852–0.904, *P* < 0.001), as shown in Figure 1.

**Table 8:** Risk factors for depression (CES-D scores) identified by multivariable logistic regression analysis (*n* = 663).

CES-D	No. of cases/no. Total cases (%)	Univariate OR (95%CI)	*P*-value	Adjusted OR (95%CI)^#^	*P*-value
**Age**
≤29	35/128	Reference			
30–35	130/374	1.416(0.909–2.205)	0.124		
≥36	60/161	1.579(0.954–2.611)	0.075		
**BMI**
<18.5	16/32	Reference			
18.5–24	131/416	0.460(0.223–0.947)	0.035^*^		
≥24	78/215	0.569(0.270–1.201)	0.139		
**Education**
Primary and Junior Secondary	37/81	1.904(1.172–3.093)	0.009^**^		
Senior Secondary and Junior College	63/174	1.285(0.884–1.868)	0.189		
Bachelor’s degree or above	125/408	Reference			
**Length of marriage**
<3	52/163	Reference			
3–5	93/278	1.073(0.710–1.622)	0.738		
>5	80/222	1.203(0.784–1.846)	0.399		
**Monthly household income**
<5,000	25/55	2.129(1.188–3.813)	0.011^*^	2.361(1.095-5.092)	0.028^*^
5,000-9,999	108/281	1.595(1.135–2.241)	0.007^**^	1.613(1.036–2.513)	0.034^*^
≥10,000	92/327	Reference		Reference	
**Pregnancy loss**					
2–3	172/554	Reference		Reference	
≥4	53/109	2.102(1.386–3.188)	<0.001^***^	2.268(1.300–3.956)	0.004^**^
**Interval since last miscarriage**					
<6	173/554	2.009(1.325–3.047)	0.001^**^	2.154(1.246–3.726)	0.006^**^
≥6	52/109	Reference		Reference	
**Pregnancy loss after 14 weeks**
Yes	46/117	1.328(0.880–2.005)	0.177		
No	179/546	Reference			
**Live birth**
Yes	10/41	0.611(0.294–1.269)	0.186		
No	215/622	Reference			
**PSQI**
<6	96/456	6.202(4.326–8.891)	<0.001^***^	5.523(3.542–8.614)	<0.001^***^
≥6	129/207	Reference		Reference	
**Active smoking**
Yes	21/59	1.084(0.620–1.895)	0.778		
No	204/604	Reference			
**Passive smoking**
Yes	91/273	0.955(0.689–1.325)	0.784		
No	134/390	Reference			
**Alcohol drinking**
Yes	40/101	1.336(0.864–2.066)	0.192		
No	185/562	Reference			
**S-AI**
<40	22/263	Reference		Reference	
≥40	203/400	11.288(6.995–18.215)	<0.001^***^	2.466(1.282–4.744)	0.007^*^
**T-AI**
<40	24/320	Reference		Reference	
≥40	201/343	17.458(10.931–27.882)	<0.001^***^	8.925(4.911–16.220)	<0.001^***^

# Adjusting age, BMI, duration of marriage, smoking status, and drinking status.

^*^P < 0.05; ^**^P < 0.01; ^***^P < 0.001.

## Discussion

Recurrent pregnancy loss is a special group of people with fragile psychology. In RPL women, feelings of guilt and loss of control were predominant (Slot et al., 2022). Moreover, women with a single pregnancy loss also experienced a significantly higher level of depression and anxiety. Studies have found that the level of anxiety differed between pregnant women who had experienced a single miscarriage and those who had experienced recurrent miscarriages. The anxiety level of pregnant women who experienced one miscarriage was markedly elevated until the week of gestation of the prior pregnancy loss and then decreased after passing this critical window of time, while the anxiety level of RPL pregnant women continued to increase (Ridaura and Raich, 2017). Either depression or anxiety alone could increase the risk of subsequent RPL, and they had a synergistic effect after the first miscarriage which increased the development of subsequent RPL disease (Wang et al., 2021). Most previous studies on psychological adjustments in RPL women had relatively small sample sizes, and sociological factors and maternal history factors are not combined. In order to better understand the psychological status of RPL women, we used multiple scales and combined social factors and maternal history factors to investigate the psychological status of RPL women, and the research results can be corroborated with each other. One of the most important findings of the present study was the high occurrence of state anxiety (60.3%), trait anxiety (51.7%), depression symptoms (33.9%), and sleep disturbance (31.2%) in women with a history of recurrent pregnancy loss. The prevalence of depression and anxiety symptoms found in the present study was much higher than those found in low-risk pregnant women (Dennis et al., 2017). And it’s illustrated that RPL women showed high psychological symptoms in eight aspects: obsessive–compulsive disease, interpersonal sensitivity, depression, anxiety, hostility, phobia, paranoia, and psychosis in the SCL-90 scale. Therefore, attention should be paid to the mental state assessment and early intervention of patients with recurrent pregnancy loss.

Among breast cancer patients, there are differences in their mental states after diagnosis, before surgery, after surgery and before chemotherapy (Li and Lin, 2016). The same goes for RPL women, who are eager to seek medical consultation, hoping to solve all problems at one time. Specifically, they hope to be able to identify the cause of the disease and treat it in a very short period of time to achieve a successful pregnancy. While it takes a long time to screen the etiology, so there are psychological fluctuations in the process of diagnosis and treatment. By comparing the anxiety and depression levels of RPL women at the first visit before pregnancy and the treatment after finding out the cause, we found for the first time that the S-AI and T-AI scores decreased and anxiety symptoms were significantly relieved after finding out the cause. The reason may be that in the initial stage of diagnosis and treatment, the unknown cause of recurrent miscarriage and the success of pregnancy preparation may have an impact on psychology as a stress stressor (Richardson et al., 2017). Comprehensive systematic etiological screening is necessary, since blind treatment because of non-standard screening in the medical treatment process of the previous patient after miscarriage will lead to the recurrence of miscarriage and aggravated anxiety. Therefore, it is necessary to strengthen the evaluation of mental state of RPL women at all stages, especially at the pre-diagnosis stage. Phased intervention and effective psychological counseling should be provided in the screening stages. It is helpful to increase the patients’ understanding and knowledge of the diagnosis and treatment process, relieve the anxiety in the process of etiological screening, and increase the compliance.

Logistic regression results showed that a bachelor’s degree or less was a risk factor for anxiety, and a monthly household income of less than 10,000 was a risk factor for depression. Patients with different educational levels have different personal expectations, social opportunities, family and social pressure, understanding of disease, and compliance with doctors (He et al., 2019). Low educators may have low compliance, especially in the treatment of recurrent miscarriage, when examinations and treatments have to be rigorous during a certain period of the menstrual cycle. Similarly, the treatment of RPL consumes huge financial resources and experiences, and stressful economic events may be associated with adverse pregnancy outcomes by activating mechanisms such as inflammation and the endocrinal system (Bruckner et al., 2016). The incidence of depression decreased with the prolongation of miscarriage interval. 26.8% of the patients scored high on the BDI immediately, 18.4% at 3 months, 16.4% at 6 months, and 9.3% at 1 year after miscarriage (Lok et al., 2010). Consistent with the above findings, in our study, the interval less than 6 months from the last miscarriage was an independent risk factor for anxiety and depression. Toffol et al. demonstrated that a high number of miscarriages was associated with an increased risk of psychiatric disorders, worsening state of mood (i.e., higher GHQ scores, increased risk of anxiety symptoms, such as fearful thoughts), and lower emotional state for certain BDI and GHQ items (Toffol et al., 2013). Consistent with the findings of this study, the higher the number of miscarriages, the higher the incidence of depression in RPL women. In the present study, 41.8% of the women had three or more previous miscarriages, and 15.3% of the women had experienced four or more previous miscarriages. This may be one of the reasons which contribute to this high prevalence of anxiety and depressive symptoms. Therefore, RPL women with such characteristics should be evaluated at multiple time points to prevent possible negative emotions such as anxiety and depression.

In this study, 14 weeks was used as the cut-off point, we found that a history of pregnancy loss after 14 weeks was a risk factor for state anxiety. Consistent with previous studies that anxiety and depression in women who experienced intrauterine death in the third trimester persisted during pregnancy until nearly 3 years after delivery (Blackmore et al., 2011). Pregnancy loss occurs at a time at which a new life is expected, and there may be no visible child, memories, or shared experiences, while the psychological impact may be greater after the second trimester when fetal movement and heart rate are gradually felt (Couto et al., 2009). Pregnancy loss in the late months reduces the expectation of pregnant women for a successful pregnancy. For such patients, more attention should be paid to their psychological status. And a history of live birth reduces the incidence of trait anxiety, consistent with the results of former studies which found that women who were involuntarily childless were more likely to be psychologically distressed with complicated grief and perceived lack of social support (Lechner et al., 2007). While a healthy child can provide some social support at the family level, social support is a buffer of negative emotions, which can alleviate anxiety and depression in women with RPL.

Mevorach-Zussman et al. (2012) explored the relationship between anxiety, quality of life, and sleep quality in 39 women with recurrent miscarriages and found that all the patients revealed a mild to moderate level of anxiety, low numbers of physical and mental health but reasonably normal values of the global quality of sleep. The incidence of sleep disturbance was 31.2% in this study, and the overall mean sleep was 4.63, which was within the normal range. Okun et al. (2018) said that poor sleep quality increases symptoms of anxiety and depression in postpartum women. Similarly, in our study, sleep disturbance is a risk factor for anxiety and depression in RPL women. Sleep patterns and sleep midpoint are closely related to negative emotions, and circadian rhythm disruptions may account for the increased risk of mood disorders (Sharkey et al., 2013). Circadian disruption may be responsible for the increased risk of mood disorders in RPL women. As the study noted, anxiety symptoms were uniquely associated with increased sleep latency and decreased sleep quality, whereas depression symptoms were uniquely associated with higher levels of daytime dysfunction, depression symptoms were associated with daytime dysfunction (Yu et al., 2016).

Another important finding of the study was that anxiety predicted depressive symptoms. In line with previous research, anxiety disorders often precede depression disorders (Kessler et al., 1996). Furthermore, evidence confirms a significant amount of overlap between anxiety and depressive symptoms amongst the general population of the childbearing women (Falah-Hassani et al., 2017). Anxiety and depressive symptoms may affect each other in pregnant women with a history of recurrent pregnancy loss. State anxiety belongs to a psychological state of tension that occurs in a certain context for a short period and with variable intensity. Trait anxiety belongs to a tendency to anxiety that is frequent or a more stable, continuous state (Knowles and Olatunji, 2020). There was a significant positive correlation between anxiety and depression symptoms in RPL women. Research evidence also suggests that trait anxiety may be associated with depression (Weger and Sandi, 2018). Further integrating anxiety scores into Logistic regression, we found that anxiety can be used as a predictor of depression, suggesting that screening anxiety symptoms in RPL women is helpful to early identify depression and prevent future depression, and strengthen psychological support for RPL women at the same time.

### Limitations and strengths

A few limitations are acknowledged. First, the current research adopts the psychological scale standard cut-off value. RPL women, as a special population, needs to be combined with clinical psychological symptoms to establish an appropriate psychological cut-off value. Second, different patients should be grouped according to the time interval of identifying etiology, and the changes of psychological state were compared. The conclusion was that the influence of identifying etiology time on mental state was concluded. Third, more data should be collected in the future to conduct longitudinal studies comparing psychological changes in RPL women at different stages of pregnancy before and after treatment. This study has the advantage of a large sample size. A variety of psychological scales were used, and the results were consistent with each other. It integrates social demographic characteristics and maternal history, discusses the psychological changes in the process of pregnancy preparation among non-pregnant women. The purpose is to detect psychological problems at the initial stage of diagnosis and treatment as early as possible.

### Conclusion

In conclusion, RPL women are a vulnerable population with a high risk for developing anxiety and depressive symptoms, which have different levels of anxiety and depression at different stages of diagnosis and treatment, so it is necessary to conduct a multi-time assessment. Education level, monthly household income, interval since last miscarriage, history of pregnancy loss after 14 weeks, and sleep disturbance are closely related to anxiety and depression, and anxiety is also a predictor of depression. Our study explored the correlation between anxiety and depression and established a model to predict depression in women with RPL, to provide a reference for the prevention and intervention of abnormal psychology in women with RPL. This study highlights the need for early identification and treatment of anxiety and depression symptoms in women with RPL. Health care professionals should make greater efforts to strengthen the social support of these women to reduce their symptoms of anxiety and depression.

## Data availability statement

The raw data supporting the conclusions of this article will be made available by the authors, without undue reservation.

## Ethics statement

The studies involving human participants were reviewed and approved by the Ethics Committee of China Medical University (approval number 2018PS381K). The patients/participants provided their written informed consent to participate in this study.

## Author contributions

YH and T-tW, and CQ conceived, designed, and initiated the study. T-tW and Y-lL contributed to the planning and implementation of the study. T-tW analyzed survey data and interpreted the results. T-tW and Y-lL drafted the manuscript. CQ and J-pL revised the manuscript. All authors contributed to the article and approved the submitted version.

## Funding

The current study was supported by grants from the National Key R&D Program of China (2016YFC1000404), the National Natural Science Foundation of China (General Program; 81370735, 81771610), the Outstanding Scientific Fund of Shengjing Hospital (201706), Science and Technology Project of Shenyang (grant number: 20-205-4-004); Leading Talents of Talent Project (grant number: XLYC2005008), Livelihood Science and Technology Joint Project of Liaoning Province (grant number: 2021JH2/10300123). These funding bodies played a role in protocol development and did not play any role in data collection, analysis, interpretation of data, or writing the manuscript.

## Conflict of interest

The authors declare that the research was conducted in the absence of any commercial or financial relationships that could be construed as a potential conflict of interest.

## Publisher’s note

All claims expressed in this article are solely those of the authors and do not necessarily represent those of their affiliated organizations, or those of the publisher, the editors and the reviewers. Any product that may be evaluated in this article, or claim that may be made by its manufacturer, is not guaranteed or endorsed by the publisher.

## Abbreviations

RPL: Recurrent pregnancy loss
BMI: Body mass index
STAI: State-trait anxiety scale
CES-D: Center for epidemiological survey, depression scale
PSQI: Pittsburgh sleep quality index
SCL-90: Symptom self-rating Scale
